# A real-time rural domestic garbage detection algorithm with an improved YOLOv5s network model

**DOI:** 10.1038/s41598-022-20983-1

**Published:** 2022-10-07

**Authors:** Xiangkui Jiang, Haochang Hu, Yuemei Qin, Yihui Hu, Rui Ding

**Affiliations:** Xi’an University of Posts and Telecommunications, Xi’an, 710121 China

**Keywords:** Agroecology, Electrical and electronic engineering

## Abstract

An increasing number of researchers are using deep learning technology to classify and process garbage in rural areas, and have achieved certain results. However, the existing garbage detection models still have problems such as high complexity, missed detection of small targets, low detection accuracy and poor real-time performance. To address these issues, we train a model and apply it to garbage classification and detection in rural areas. In general, we propose an attention combination mechanism based on the YOLOv5 algorithm to build a better backbone network structure, add a new small object detection layer in the head network to enhance the model's ability to detect small objects, adopt the CIoU loss function to optimize the output prediction bounding box, and choose the Adam optimization algorithm to train the model. Our proposed YOLOv5s-CSS model detects a single garbage image in 0.021 s with a detection accuracy of 96.4%. Compared with the YOLOv5 algorithm and the classic detection algorithm, the improved algorithm has better detection speed and detection accuracy. At the same time, the complexity of the network model is reduced to a certain extent, which can meet the requirements of real-time detection of rural domestic garbage.

## Introduction

Environmental pollution caused by the accumulation of domestic garbage is a serious problem in rural areas all over the world^1^. Especially in developing countries, due to the continuous development of rural economies and changes in rural residents’ lifestyles, the production of rural domestic garbage has increased significantly^2^. For example, in China, which is the largest garbage producer in the world with an 8% to 10% annual growth rate, rural domestic garbage pollution has become one of the main sources of pollution^3^. Environmental pollution has become one of the top environmental concerns in China^4^.

Due to the characteristics of many types of domestic garbage, the high moisture content in rural areas and whether domestic garbage is disposed of at a garbage incineration power plant or a garbage terminal treatment landfill, it is difficult to fully use resources and reduce disposal^5^. There are several problems with rural domestic garbage disposal through traditional methods, for instance, the reliance on manual participation, low classification efficiency and poor working environment. With the existence and spread of COVID-19, workers face hazards when they deal with garbage. How to use deep learning for effective garbage sorting has become a hot research topic. In recent years, deep learning has evolved rapidly and penetrated into various industries. A large number of results has been achieved using deep learning in search technology^6^, data mining^7^, machine learning^8^ and natural language processing^9^. Therefore, using deep learning techniques provides a new solution to the garbage classification problem in rural areas.

Ma et al.^10^ proposed an improved Faster R-CNN (Faster Regions with CNN features) algorithm. First, the Faster R-CNN algorithm is combined with VGG (Visual Geometry Group Network)-16 and ResNet (Residual Network)-50 convolutional neural networks to improve the detection accuracy of small objects. Second, the Soft-NMS algorithm is used to replace the traditional nonmaximum suppression algorithm, and the parameters are analyzed to determine the parameter range. Finally, garbage detection is realized. It still takes 4.103 s to detect a single image, and the detection speed needs to be improved. Wang Hao^11^ built a VGG-16 convolutional neural network to solve the problem of domestic garbage detection and classification. First, a computer vision library was used to locate and select recognized objects, and preprocess the image. Second, the ReLU (Rectified Linear Unit) activation function is used to increase the BN (Batch Normalization) layer to improve the recognition accuracy of the model and accelerate the convergence speed of the model. Finally, the detection accuracy of domestic garbage is only 75.2%, and the detection accuracy needs to be strengthened. Wu Han^12^ developed a lightweight garbage detection model MobileNetV3_Lite. First, the characteristics of the lightweight structure of the MobileNetV3 module are analyzed. Second, based on the YOLO (You Only Look Once) v3 detection algorithm, the MobileNetV3 module is embedded in the backbone network of the algorithm to construct a lightweight garbage detection model. Finally, the real-time performance of the model is tested, with only 25 frames per second. Although the model is lightweight, it cannot meet the requirements of real-time detection. Wu et al.^13^ designed a GC-YOLOv5 garbage detection model. First, the garbage images are preprocessed to obtain a garbage dataset. Second, the model is trained based on the YOLOv5 algorithm. Finally, only five types of household garbage can be detected. The limitation of this model is that there are fewer garbage categories detected, and its practicality needs to be improved.

Rabano et al.^14^ developed a MigeNet detection model based on the TensorFlow framework. First, they collect and create a dataset of garbage images. Second, the model is trained 500 times using the transfer learning technique. Finally, the model is tested and analyzed. The accuracy of the model is only 87.2%, and it is necessary to continue to optimize the accuracy of the model to detect garbage. Rismiyati et al.^15^ used transfer learning techniques to focus on pretrained models such as VGG-16, ResNet-50 and Xception. The validation accuracy of Xception on the dataset is 88%. Kumar et al.^16^ proposed a YOLOv3-based approach to efficiently separate disposal and recycling garbage. The YOLOv3 algorithm is used to train a homemade dataset. The model is successfully trained using only six object types. In addition, in the detection process, YOLOv3-tiny is utilized to verify the capability of YOLOv3. Since there are few types of detected garbage, there are certain limitations in its actual use. Seredkin et al.^17^ first, trained a model on nearly 13,000 canned garbage images based on a CNN (Convolutional Neural Network) model. Second, the canned garbage is transferred to the conveyor belt. Finally, the CNN-based model classifies the target object, and the classification accuracy is only 64%. Although the model uses a large number of garbage images for training, the learning effect based on the CNN model is poor. Wieczorek et al.^18^ developed a lightweight CNN architecture to achieve lightweight improvements to the model by using a minimum number of processing layers, and designed a new sliding window procedure. However, the method suffers from the problem of tedious process of detecting target objects when performing detection tasks. Marcin et al.^19^ propose a novel correlation learning mechanism (CLM) for deep neural network architectures that combines convolutional neural network (CNN) with classic architecture. The proposed model of correlation learning mechanism is composed of convolutional neural network coworking in training process with classic neural network. This learning mechanism requires the researcher’s experience to select the filter size and continuously adjust the parameters to obtain the best fitting process for better learning efficiency and accuracy. Therefore, this learning mechanism lacks some adaptive update function. Hussain et al.^20^ proposed a Revise-Net model that efficiently classifies the boundary pixels using a combination of binary cross-entropy, similarity index, and intersection over union losses at the pixel, patch, and map levels, thereby effectively segmenting the saliency objects in an image. Therefore, the Revise-Net model is only used for image segmentation, separating the target object from the background of the current image, and cannot detect the target object in the video.

The above methods have made certain contributions in the field of garbage classification, but there are still three problems to be solved: (1) In the face of a complex network structure, many computing resources and high-cost model training are needed; (2) Small objects are detected; and (3) In practical applications, not only the requirements of high detection accuracy should be met but also, the principle of real-time performance should be taken into account. For the first problem, we adopt the YOLOv5s model as the baseline and introduce the CBAM (Convolutional Block Attention Module) attention module. The YOLOv5s model has a simple structure, and the model size is only 14.4 MB. At the same time, the CBAM attention module does not have a large number of convolution structures; therefore, the lager number of calculations caused by convolution multiplication is avoided, making the model complexity low and the amount of calculation small. For the second problem, we add a small object detection layer to the output prediction part of the YOLOv5s model to construct a new output prediction network. By adding a new set of anchor box values, the features of small objects are extracted, and a four-layer output prediction network is constructed to meet the needs of small object detection. For the third problem, we propose an attention combined mechanism to enhance the ability of the model to extract features and optimize the network structure to improve the real-time detection. In the process of training the model, there are problems such as slow convergence speed and fluctuation of the loss value. We introduce the Adam (Adaptive Moment Estimate) optimization algorithm to solve this problem. When outputting the prediction results, there is a problem that the gap between the prediction bounding box and the ground truth bounding box is too large. We optimize the output prediction bounding box by changing the loss function type to make it closer to the ground truth bounding box.

Our proposed model is a real-time lightweight YOLOv5 architecture for fast detection of domestic garbage in rural areas. The model can effectively detect domestic garbage in complex scenes. In these scenarios, garbage images may be difficult to detect due to blurring or other conditions, and the proposed model can be well applied in similar scenarios. The novelty of our model lies in the proposed YOLOv5 architecture, in which an attention combination mechanism is introduced to enhance the model's ability to extract features and optimize the network structure to improve real-time detection. A new output prediction network is constructed in the output prediction part of the model to meet the needs of small target object detection. The whole model was trained by using the Adam algorithm, as this one was the most effective in our research tests. Finally, the output prediction bounding box is optimized by changing the loss function of the model. Therefore, typical rural domestic garbage is taken as the research object in this paper, thirteen types of garbage are collection, and a garbage dataset is made to solve the above three problems. The results show that the calculation volume of the YOLOv5s-CSS model is reduced by 45.3%, the detection accuracy is increased by 4.6%, the inference time is shortened by 7.4 ms, and the FPS is as high as 47.6 frames/s. It can identify multiple types of garbage at the same time, provide technical solutions for the intelligent disposal of rural garbage, and realize real-time detection.

This paper is structured as follows: In the “Target detection algorithms” section, the mainstream target detection algorithms in the field of target recognition and the algorithm used in this study primarily highlighted. In the “Related improvement work” section, the improvements made work in this study are explained, and a YOLOv5s-CSS garbage detection model is established. In the “Experimental results and analysis”, the experimental procedure, experimental results, and experimental analysis of the improvement work is explained. In the “Discussion” section, the feasibility of the proposed method is discussed. In the “Conclusion and future work” section, the contributions and content of this study are summarized.

## Target detection algorithms

There are two main types of target detection algorithms, which are traditional target detection algorithms and target detection algorithms based on deep learning. Most of the traditional target detection algorithms are based on sliding windows and artificial feature extraction, which have the disadvantages of high computational complexity and poor robustness in complex scenes. Compared with traditional algorithms, based on deep learning target detection algorithm has the advantages of fast speed, high accuracy and strong robustness under complex conditions.

Target detection algorithms based on deep learning are mainly divided into two types: algorithms based on regression, and algorithms based on localization and classification. Target detection algorithms based on regression are also called one-stage target detection algorithms. These algorithms do not generate target regions directly but consider the target detection task as a regression of the whole image. The mainstream one-stage target detection algorithms are YOLO^21^ and SSD (Single Shot MultiBox Detector)^22^. The target detection algorithm based on localization and classification are also called two-stage target detection algorithms, which divide the target detection problem into two stages: in the first stage, candidate region localization is generated, and in the second stage, prediction results are generated from the target region after feature extraction, classification and regression. Representative two-stage target detection algorithms include Faster R-CNN^23^ and others.

The two-stage object detection algorithm generates candidate regions in the first stage and classifies and regresses only the content of the region of interest in the second stage, losing the spatial information of local objects in the whole image. For this reason, single-stage object detection algorithms can solve this drawback. Compared with SSD single-stage object detection algorithm, YOLO series algorithm shows good detection performance with smaller network structure, and YOLOv5 algorithm is the latest proposed object detection algorithm of YOLO series algorithm. YOLOv5 algorithm has surpassed the other four versions of YOLO series algorithm in terms of detection speed and detection accuracy, and has better performance. These four versions are YOLOv1, YOLOv2, YOLOv3 and YOLOv4. Therefore, we choose YOLOv5 as the main algorithm for this research.

### YOLOv5 algorithm

**Figure 1:**
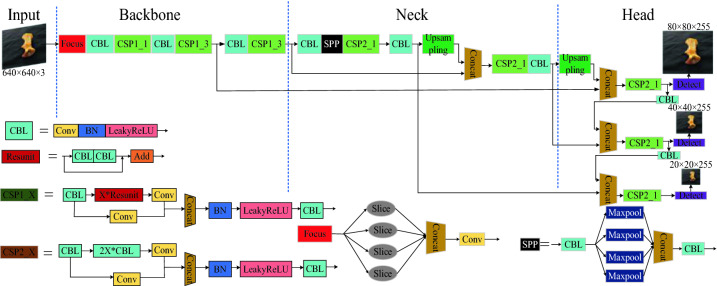
YOLOv5 algorithm network model structure.

YOLOv5^24^ is a one-stage target recognition algorithm proposed by Glenn Jocher in 2020. Compared with the previous four generations of the YOLO series of algorithms, the YOLOv5 algorithm contains four pretrained models, named YOLOv5s, YOLOv5m, YOLOv5l and YOLOv5x. The model volume size can be changed by modifying the parameters of the width and depth of the pretrained model. The parameters of model size, width and depth for different pretrained models are shown in Table 1. The YOLOv5s framework mainly consists of three components: the backbone network, neck network and head network. The network structure is shown in Fig. 1.

**Table 1:** Summary of the network model parameters for the YOLOv5 algorithm.

Parameter categories	YOLOv5s	YOLOv5m	YOLOv5l	YOLOv5x
Width	0.50	0.75	1.00	1.25
Depth	0.33	0.67	1.00	1.33
Model size	14.4 MB	41.1 MB	90.1 MB	167 MB

### Pretraining network models

**Figure 2:**
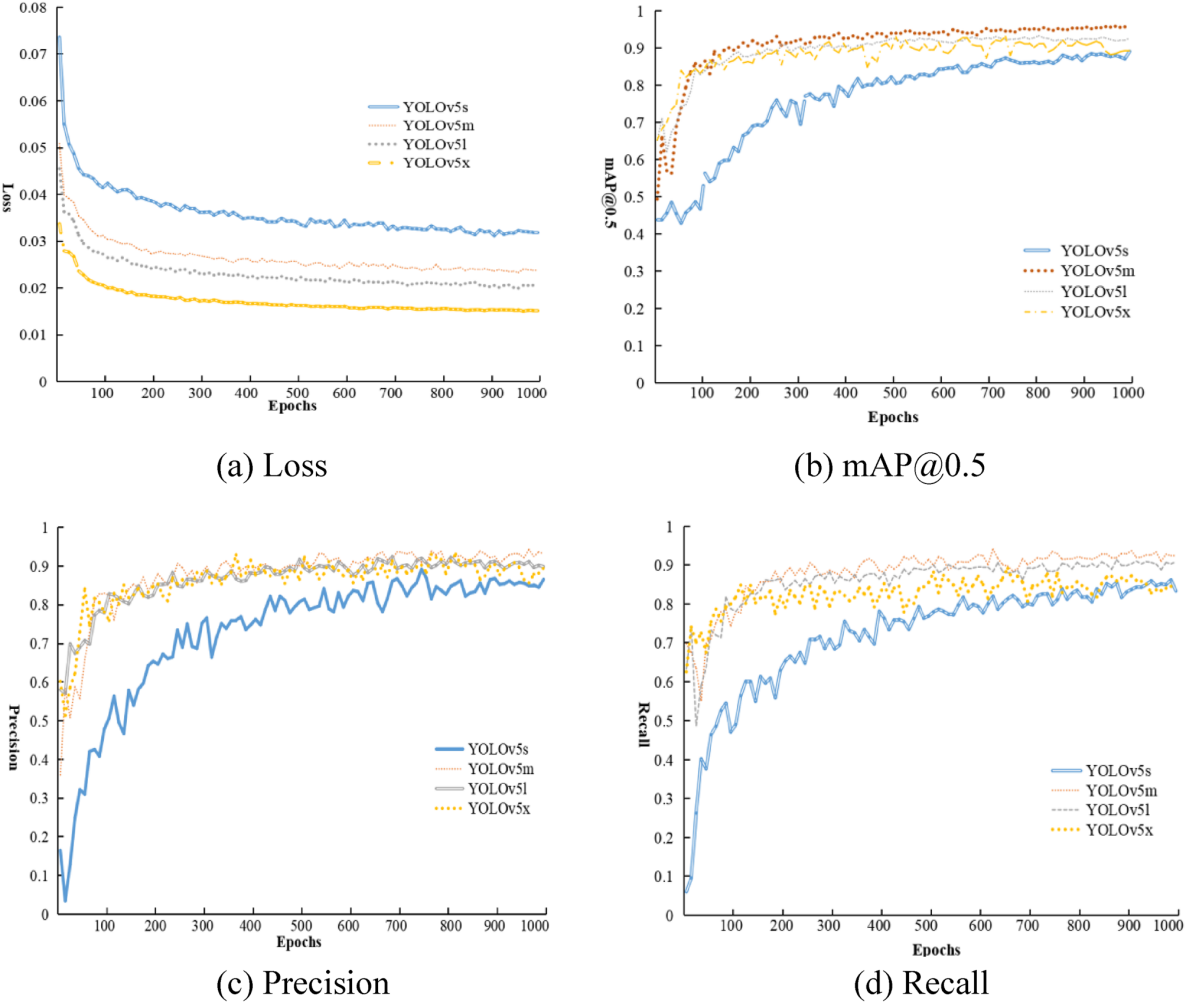
Training process of four pretrained models.

The YOLOv5 target detection algorithm provides four pretrained models, named YOLOv5s, YOLOv5m, YOLOv5l, and YOLOv5x. First, these four models are trained for 1000 epochs based on the garbage dataset introduced in Section “Dataset”. Second, the training process is recorded among the four performance metrics, the loss function, mAP@0.5, precision and recall. Finally, the performance metrics of the four pretrained models are compared. Figure 2 shows the training process of the models. The comparison of the corresponding performance metrics of the models is shown in Table 2.

**Table 2:** Comparison of the performance metrics for the four pretrained models.

Model	Precision (%)	Recall (%)	mAP@0.5 (%)	Inference time (ms)	Model size (MB)
YOLOv5s	91	88.1	91.8	19.8	14.4
YOLOv5m	94	93.9	96	70.1	42.8
YOLOv5l	92.7	90.4	92.9	122.9	89.4
YOLOv5x	90.3	86.6	91.9	306.1	169.5

Table 2 shows the performance index analysis of the above four pretrained models. In terms of the garbage dataset used in this study, the accuracy of the YOLOv5m model is the highest. However, the YOLOv5m network model has a long inference time, large model volume, and high model complexity. It also requires high computational cost; therefore, it cannot meet the requirements of real-time performance. Although the accuracy of the YOLOv5s model is lower than that of the other three models, it still remains above 91%. The YOLOv5s model has the shortest inference time, a smaller model size, and low model complexity. It can not only save calculation, costs but also meet the requirements of real time. Therefore, according to the actual requirements, we choose the YOLOv5s network model and improve it.

## Related improvement work

### Attention combination mechanism

**Figure 3:**
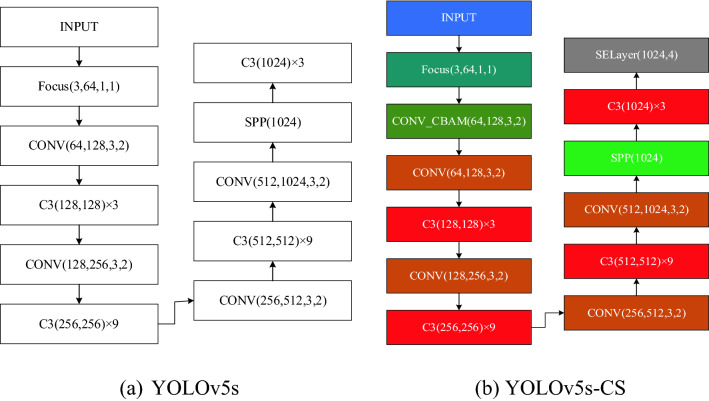
YOLOv5 backbone network structure before and after improvement.

Due to the difficulty in extracting features from target areas in images, the high computational effort of the model and the low accuracy of detection are addressed. As shown in Fig. 3, we introduce a lightweight feedforward convolutional attention module CBAM after the backbone network Focus module of the YOLOv5s network model. The SE-Net (Squeeze and Excitation Networks) channel attention module is posted at the end of the backbone network. We propose an attention combination mechanism based on the YOLOv5s network model and name the improved network model YOLOv5s-CS. Where the CBAM module has a channel number of 128, a convolutional kernel size of 3 and a step size of 2, the SELayer has a channel number of 1024 and a step size of 4.

#### Convolutional block attention module network

**Figure 4:**
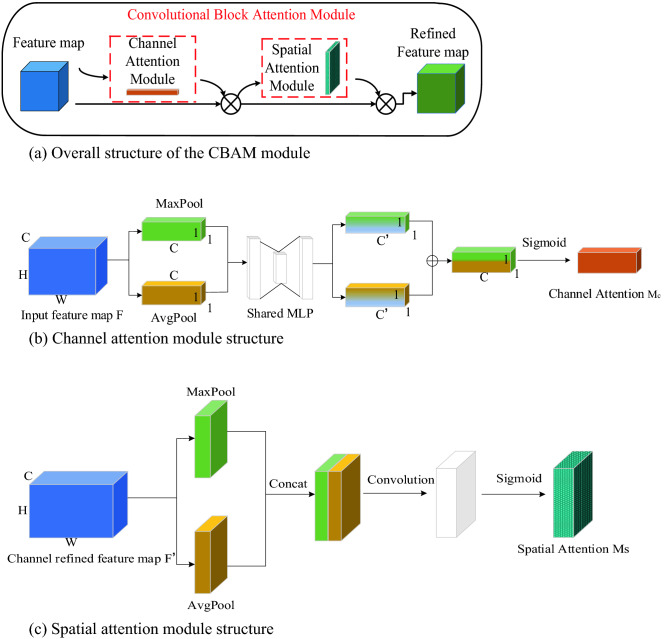
Principle of CBAM.

In 2018, Woo et al.^25^ proposed the lightweight feedforward convolutional attention module CBAM. The CBAM module focuses on feature information from both channels and space dimensions and combines feature information to some extent to obtain more comprehensive reliable attentional information^26^. CBAM consists of two submodules, the channel attention module (CAM) and spatial attention module (SAM), and its overall module structure is shown in Fig. 4a.

The working principle of the CAM is shown in Fig. 4b. First, the feature map F is input at the input entrance. Second, the global maximum pooling operation and the global average pooling operation are applied to the width and height of the feature map respectively to obtain two feature maps of the same size. Third, two feature maps of the same size are input to the shared parameter network MLP at the same time. Finally, the new feature map output from the shared parameter network is subjected to a summation operation and a sigmoid activation function to obtain the channel attention features ${M}_{c}$.

The channel attention module CAM is calculated as shown in Formula (1):

1 $${\text{M}}_{\rm{c}}({\text{F}}){=\sigma}({\text{MLP (AvgPool (F))}}+ {\text MLP (MaxPool (F)))}{=\sigma}({\rm{W}}_{1}({\text{W}}_{0}({\text{F}}_{{{\rm{avg}}}^{\rm{c}}}))+{\rm{W}}_{0}({\rm{W}}_{1}({\rm{F}}_{{{\rm{max}}}^{\rm{c}}})))$$

where *σ* represents the sigmoid function, *MLP* represents the shared parameter network, ${\text{W}}_{0}$ and ${\text{W}}_{1}$ represent the shared weights, ${\text{F}}_{\text{avg}}^{\text{c}}$ is the result of feature map F after global average pooling, and ${\text{F}}_{\text{max}}^{\text{c}}$ is the result of feature map F after global maximum pooling.

The working principle of SAM is shown in Fig. 4c. The feature map F' is regarded as the input of the SAM. F' is obtained by multiplying the input of SAM with the output of CAM. First, the global maximum pooling operation and the global average pooling operation are applied to the channels of the feature map to obtain two feature maps of the same size. Second, two feature maps that have completed the pooling operation are stitched at the channels and the feature channels are dimensioned down using the convolution operation to obtain a new feature map. Finally, spatial attention features ${\text{M}}_{\text{s}}$ are generated using the sigmoid activation function.

The spatial attention module (SAM) is calculated, as shown in Formula (2):

2 $${\text{M}}_{\text{s}}\left({\text{F}}\right) {=\sigma}\left({\text{f}}^{7 \times 7}\left(\left[{\text{AvgPool}}\left({\text{F}}\right)\text{;MaxPool}\left({\text{F}}\right)\right]\right)\right) {=\sigma}\left({\text{f}}^{7 \times 7}\left(\left[{\text{F}}_{\text{avg}}^{\text{s}} ; {\text{F}}_{\text{max}}^{\text{s}}\right]\right)\right)$$

where *σ* is the sigmoid function, ${\text{f}}^{7 \times 7}$ denotes the convolution operation with a filter size of 7 × 7, ${\text{F}}_{\text{avg}}^{\text{s}}$ is the result of the feature map after global average pooling, and ${\text{F}}_{\text{max}}^{\text{s}}$ is the result of the feature map after global maximum pooling.

#### Squeeze and excitation network

In 2018, Hu et al.^27^ proposed a single-path attention network structure SE-Net. SE-Net uses the idea of an attention mechanism to analyze the relationship feature maps by modeling and adaptively learning to obtain the importance of each feature map^28^ and then assigns different weights to the original feature map for updating according to the importance. In this way, SE-Net pays more attention to the features that are useful for the target task while suppressing useless feature information and allocates computational resources rationally to different channels to train the model to achieve better results.

**Figure 5:**
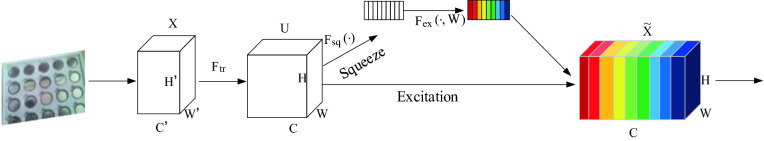
The SE-Net module structure.

The SE-Net attention module is mainly composed of two parts: the squeeze operation and excitation operation. The structure of the SE-Net module is shown in Fig. 5.

The squeeze operation uses global average pooling to encode all spatial features on the channel as local features. Second, each feature map is compressed into a real number that has global information on the feature maps. Finally, the squeeze results of each feature map are combined into a vector as the weights of each group of feature maps. It is calculated as shown in Eq. (3):

3 $${\text{Z}}_{\text{c}}={\text{F}}_{\text{sq}}\left({\text{u}}_{\text{c}}\right)=\frac{1}{\text{H} \times {\text{W}}}\sum_{\text{i=1}}^{\text{H}}\sum_{\text{j=1}}^{\text{W}}{{\text{u}}}_{\text{c}}\left(\text{i,j}\right) \, \, \, $$

where *H* is the height of the feature map, *W* is the feature map width, *u* is the result after convolution, *z* is the global attention information of the corresponding feature map, and the subscript *c* indicates the number of channels.

After completing the squeeze operation to obtain the channel information, the feature vector is subjected to the excitation operation. First, it passes through two fully connected layers. Second, it uses the sigmoid function. Finally, the output weights are assigned to the original features. It is calculated as follows:

4 $$\text{s} = {\text{F}}_{\text{ex}}\left(\text{z,W}\right){=\sigma}\left({\text{g}}\left(\text{z,W}\right)\right){=\sigma}\left({\text{W}}_{2}{\delta}\left({\text{W}}_{1}{\text{z}}\right)\right)$$

5 $$\widetilde{{\text{x}}_{\rm{c}}}={\text{F}}_{\rm{scale}}\left({\text{u}}_{\rm{c}}, {\text{s}}_{\rm{c}}\right)={\text{s}}_{\rm{c}}{{\text{u}}}_{\rm{c}}$$

where *σ* is the ReLU activation function, *δ* represents the sigmoid activation function, and ${\text{W}}_{1}$ and ${\text{W}}_{2}$ represent two different fully connected layers. The vector *s* represents the set of feature mapping weights obtained through the fully connected layer and the activation function. $\widetilde{{x}_{c}}$ is the feature mapping of the x feature channel, ${\text{s}}_{\text{c}}$ is a weight, and ${\text{u}}_{\text{c}}$ is a two-dimensional matrix.

### Target detection layer

**Figure 6:**
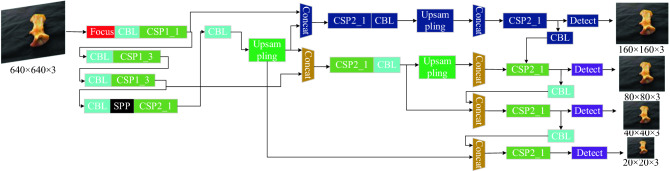
The YOLOv5s-STD network structure.

The garbage in rural areas is a smaller target and has fewer pixel characteristics, such as capsule, button butteries. Therefore, we insert a small target detection layer to improve the head network structure based on the original YOLOv5s network model for detecting objects with small targets to optimize the problem of missed detection in the original network model. The YOLOv5s network structure with the addition of the small target detection layer is shown in Fig. 6 and named YOLOv5s-STD.

In the seventeenth layer of the neck network, operations such as upsampling are performed on the feature maps so that the feature maps continue to expand. Meanwhile, in the twentieth layer, the feature maps obtained from the neck network are fused with the feature maps extracted from the backbone network. We insert a detection layer capable of predicting small targets in the thirty-first layer. To improve the detection accuracy, we use a total of four detection layers for the output feature maps, which are capable of detecting smaller target objects. In addition to the three initial anchor values based on the original model, an additional set of anchor values is added as a way to detect smaller targets. The anchor values of the improved YOLOv5s network model are set to [5, 6, 8, 14, 15, 11], [10, 13, 16, 30, 33, 23], [30, 61, 62, 45, 59, 119] and [116, 90, 156, 198, 373, 326].

### Bounding box regression loss function

The loss function is an important indicator of the generalization ability of a model. In 2016, Yu et al.^29^ proposed a new joint intersection loss function IoU for bounding box prediction. IoU stands for intersection over union, which is a frequently used metric in target detection. It is used not only to determine the positive and negative samples, but also to determine the similarity between the predicted bounding box and the ground truth bounding box. It can be described as shown in the Eq. (6):

6 $$\text{IoU} = \frac{\left|\text{A} \cap\left.{\text{B}}\right|\right.}{\left|{\text{A}} \cup\left.{\text{B}}\right|\right.}$$

where the value domain of *IoU* ranges from [0,1]. *A* and *B* are the areas of arbitrary regions. Additionally, when IoU is used as a loss function, it has to scale invariance, as shown in Eq. (7):

7 $$\text{IoU\_Loss} = 1-\frac{\left|\text{A} \cap \left.{\text{B}}\right|\right.}{\left|{\text{A}} \cup \left.{\text{B}}\right|\right.}$$

However, when the prediction bounding box and the ground truth bounding box do not intersect, namely IoU = 0, the distance between the arbitrary region area of A and B cannot be calculated. The loss function at this point is not derivable and cannot be used to optimize the two disjoint bounding boxes. Alternatively, when there are different intersection positions, where the overlapping parts are the same but in different overlapping directions, the IoU loss function cannot be predicted.

**Figure 7:**
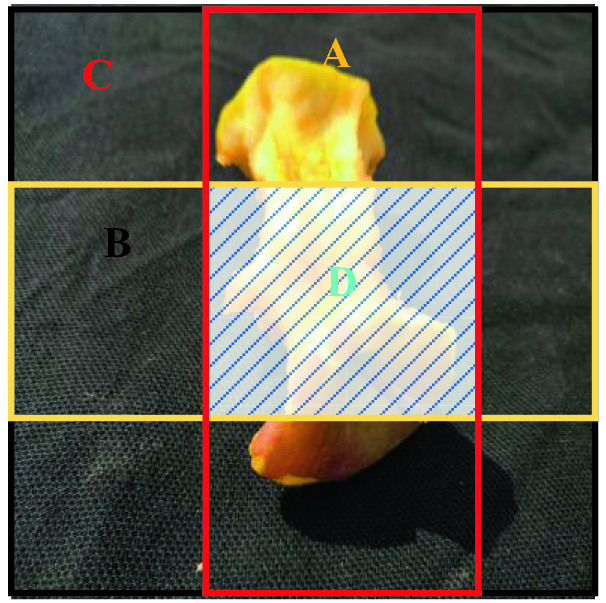
GIoU evaluation chart.

To address these issues, the idea of GIoU (Generalized Intersection over Union)^30^, in which a minimum rectangular Box C of A and B is added, was proposed in 2019 by Rezatofighi et al. Suppose the prediction bounding box is B, the ground truth bounding box is A, the area where *A* and *B* intersect is *D*, and the area containing two bounding boxes is *C*, as shown in Fig. 7.

Then, the GIoU calculation, as shown in Formula (8), is:

8 $$\text{GIoU}= \text{IoU}-\frac{\text{|C}-\left({\text{A}} \cup {\text{B}}\right)\text{|}}{\text{|C|}}$$

The GIoU_Loss is calculated as (9):

9 $$\text{GIoU\_Loss=1}-{\text{IoU}}-\frac{\text{|C}-\left({\text{A}}  \cup {\text{B}}\right)\text{|}}{\text{|C|}}$$

The original YOLOv5 algorithm uses GIoU_Loss as the loss function. Comparing Eqs. (6) and (8), it can be seen that GIoU is a new penalty term $\frac{\text{|C}-\left({\text{A}} \cup {\text{B}}\right)\text{|}}{\text{|C|}}$ that is added to IoU and is clearly represented by Fig. 7.

**Figure 8:**
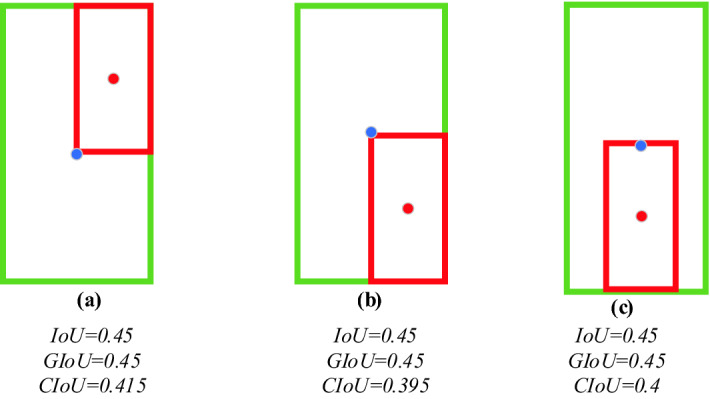
Comparsion of loss values.

Although the GIoU loss function solves the problem that the gradient of the IoU loss function cannot be updated in time and the prediction bounding box, the direction of the ground truth bounding box is not consistent when predicting, but there are still disadvantages, as shown in Fig. 8.

Figure 8 shows three different position relationships formed when the predicted bounding box and the ground truth bounding box overlap exactly. Among them, the ratio of the length to width of the green grounding truth bounding box is 1:2, and the red predicted bounding box has the same aspect ratio as the ground truth bounding box, but the size is only one-half of the green ground truth bounding box. When the prediction bounding box and the ground truth bounding box completely overlap, the GIoU degenerates to the IoU, and the GIoU value and IoU value for the three different position cases are 0.45 at this time. The GIoU loss function does not directly reflect the distance between the prediction bounding box and the ground truth bounding box. Therefore, we introduce the CIoU (Complete Intersection over Union)^31^ loss function to replace the original GIoU loss function in the YOLOv5 algorithm and continue to optimize the prediction bounding box.

Therefore, the CIoU is calculated as (10):

10 $$\text{GIoU\_Loss}=1-\text{IoU}-\frac{\text{|C}-\left({\text{A}} \cup {\text{B}}\right)\text{|}}{\text{|C|}}$$

where *b* and ${\text{b}}^{\text{gt}}$ denote the centroids of the prediction bounding box and the ground truth bounding box, respectively, ${\rho}$ is the Euclidean distance between the two centroids, and *c* is the diagonal length of the minimum closed area formed by the prediction bounding box and the ground truth bounding box.

$\alpha$ is the parameter used to balance the scale, and *v* is the scale consistency used to measure the aspect ratio between the prediction bounding box and the ground truth bounding box, as shown in Eqs. (11) and (12).

11 $$\alpha =\frac{\text{v}}{\left(1-\text{IoU}\right)+{\text{v}}^{{\prime}}}$$

12 $$\text{v} = \frac{4}{{\pi}^{2}}{\left({\text{arctan}}\frac{{\omega}^{\text{gt}}}{{\text{h}}^{\text{gt}}}- \text{arctan}\frac{{\omega}^{\text{p}}}{{\text{h}}^{\text{p}}}\right)}^{2}$$

Therefore, the expression of CIoU_Loss can be obtained according to Eqs. (10), (11) and (12).

13 $$\text{CIoU\_Loss} =1-\text{CIoU}=1-\text{IoU}+\frac{{\rho}^{2}\left(\text{b,}{\text{b}}^{\text{gt}}\right)}{{\text{c}}^{2}}{+ \alpha v }$$

### Optimization algorithm

After optimizing the loss function of the network model, the next step is to optimize the hyperparameters of the network model. The function of the optimizer is to adjust the hyperparameters to the most appropriate values while making the loss function converge as much as possible^32^. In the target detection algorithm, the optimizer is mainly used to calculate the gradient of the loss function and to iteratively update the parameters.

The optimizer used in YOLOv5 is stochastic gradient descent (SGD). Since a large number of problems in deep learning satisfy the strict saddle function, all the local optimal solutions obtained are almost as ideal. Therefore, SGD algorithm is not trapped in the saddle point and has strong generality. However, the slow convergence speed and the number of iterations of SGD algorithm are still problems that need to be improved. Adam algorithm has both the first-order momentum in the SGD algorithm and combines the second-order momentum in AdaGrad algorithm and AdaDelta algorithm, Adaptive&Momentum. Adam formula can be described as follows:

14 $${m}_{t}={\beta }_{1}{m}_{t-1}+\left(1-{\beta }_{1}\right){g}_{t}$$

15 $${v}_{t}={\beta }_{2}{v}_{t-1}+\left(1-{\beta }_{2}\right){g}_{t}^{2}$$

16 $${\widehat{m}}_{t}=\frac{{m}_{t}}{1-{\beta }_{1}^{t}}$$

17 $${\widehat{v}}_{t}=\frac{{v}_{t}}{1-{\beta }_{2}^{t}}$$

where ${\beta }_{1}$ and ${\beta }_{2}$ parameters are hyperparameters and *g* is the current gradient value of the error function, ${m}_{t}$ is the gradient of the first-order momentum and ${v}_{t}$ is the gradient of the second-order momentum.

Adam is an adaptive one-step random objective function optimization algorithm based on a low-order moment. It can replace the traditional first-order optimization algorithm for the stochastic gradient descent process. It is able to update the weights of the neural network adaptively based on the data trained during the iterative process. The Adam optimizer occupies fewer memory resources during the training process and is suitable for solving the problems of sparse gradients and large fluctuations in loss values^33^. Therefore, we use the Adam optimization algorithm instead of the SGD optimization algorithm to train the network model based on the YOLOv5s network model. The calculation is shown in Table 3.

**Table 3:** Computing method of the Adam optimizer.

Algorithm	The Adam algorithm
Step 1	Initialize the parameters ${\text{V}}_{{d\omega}}= \text{0}$*, *${\text{S}}_{{d\omega}}= \text{0}$*, *${\text{V}}_{\text{db}}= \text{0}$*,*${\text{S}}_{\text{db}}= \text{0}$
Step 2	After the *t*th iteration, given *dw* and *db* based on the minibatch gradient descent algorithm
Step 3	Calculate the weighted average sum of momentum index
	${V}_{dw}={\beta }^{{\prime}}{V}_{dw}+(1-{\beta }^{{\prime}})dw$, ${V}_{db}={\beta }^{{\prime}}{V}_{db}+(1-{\beta }^{{\prime}})db$
Step 4	Perform gradient and weight updates using the RMSprop algorithm
	${S}_{dw}={\beta }^{{\prime}}{S}_{dw}+(1-{\beta }^{{{\prime}}{^{\prime}}})d{w}^{2}$, ${S}_{db}={\beta }^{{^{\prime}}{^{\prime}}}{S}_{db}+(1-{\beta }^{{^{\prime}}{^{\prime}}})db$
Step 5	Calculate the correction and deviation values of the momentum index and RMSprop algorithm
	${V}_{dw}^{c}={V}_{dw}/(1-{\beta }^{{{\prime}}t})$*, *${V}_{db}^{c}={V}_{db}/(1-{\beta }^{{{\prime}}t})$*, *${S}_{dw}^{c}={S}_{dw}/(1-{\beta }^{{{\prime}}{^{\prime}}t})$*,*${S}_{\text{db}}^{c}={S}_{db}/(1-{\beta }^{{{\prime}}{^{\prime}}t})$
Step 6	Update the weights $\omega$ and $b$ according to the first five steps
	$\omega =\omega -\alpha ({v}_{dw}^{c}/ \sqrt{{s}_{dw}^{c}}+\varepsilon )$*,* $b=b - \alpha ({v}_{db}^{c}/ \sqrt{{s}_{db}^{c}}+\varepsilon )$

where $\alpha$ is a factor controlling the learning rate of the network, ${\beta}^{{\prime}}$ is the exponential decay rate of the first-order moment estimate, ${\beta}^{{\prime \prime}}$ is the exponential decay rate of the second-order moment estimate, and ${\varepsilon}$ is a constant that tends to zero infinitely as the denominator.

where ${\alpha}$ is a factor controlling the learning rate of the network, ${\beta}^{{\prime}}$ is the exponential decay rate of the first-order moment estimate, ${\beta}^{{\prime\prime}}$ is the exponential decay rate of the second-order moment estimate, and ${\varepsilon}$ is a constant that tends to zero infinitely as the denominator.

## Experimental results and analysis

### Model performance evaluation metrics

To evaluate the performance of the improved YOLOv5s network model for detecting garbage, in this paper, six different performance metrics are evaluated: (1) model size; (2) precision; (3) recall; (4) mAP value; (5) frames per second (FPS) and (6) computational volume. Their formulas are given as follows:

18 $$\text{Pr} = \frac{\text{TP}}{\text{TP+FP}}$$

19 $$\text{Re} = \frac{\text{TP}}{\text{TP+FN}}$$

20 $$\text{AP} = {\int }_{0}^{1}{\text{P}}\left({\text{r}}\right){\text{dr}}$$

21 $$\text{mAP} = \frac{1}{{\text{N}}}\sum_{\text{i}=1}^{\rm{N}} \, {\text{AP}}_{\rm{i}}$$

22 $$\text{FPS} = \frac{1}{{\text{t}}}$$

where, *TP* stands for true positive samples, which indicates the number of samples of garbage images correctly detected and identified. *FN* stands for false negative samples, which indicates the number of samples of garbage images incorrectly detected and identified. *FP* stands for false-positive samples, which indicates the number of garbage samples missed. *FPS* stands for the number of images that can be processed per second, and *t* stands for the time required to process the images.

### Experimental platform

The experimental environment was based on the deep learning framework PyTorch^34^ and the Windows 10 operating system. A virtual environment was created on Anaconda Navigator. Python 3.6, PyTorch 1.7.1 and Cuda 10.1 were installed in the virtual environment. The CPU was an Intel Core i5-10400F, and the GPU was an NVIDIA GeForce GTX1650. A modified YOLOv5s network structure was used for iteration training, the initial learning rate was set to 0.01, the batch size was set to 12, and the number of epochs was set to 1000.

### Dataset

**Figure 9:**
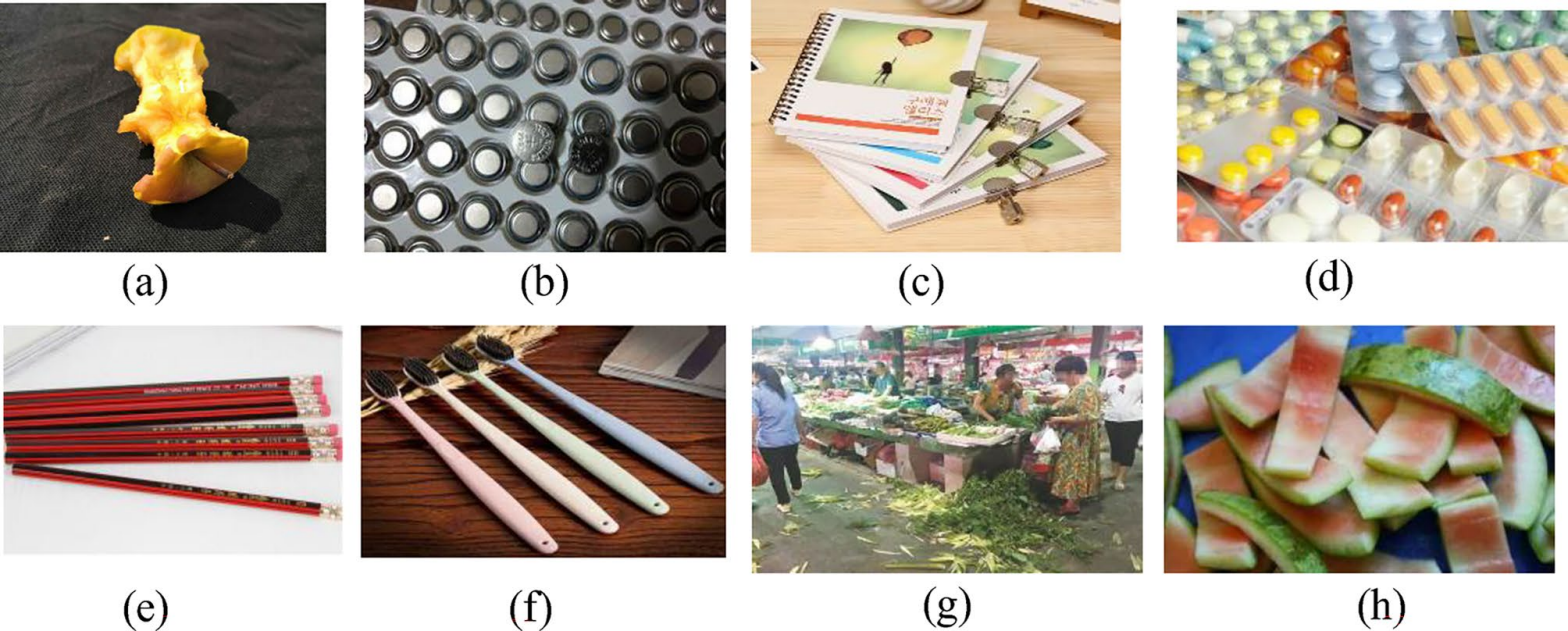
Examples of garbage images in the dataset: (**a**) apple core, (**b**) button battery, (**c**) book, (**d**) capsule, (**e**) pencil, (**f**) toothbrush, (**g**) vegetable leaf, (**h**) watermelon peel.

In this study, the type and size of garbage to be detected are identified. Garbage images are collected among rural areas through online collection, video capture of domestic garbage and shooting. Using the image annotation tool, and annotating the collected garbage images with information, a total of thirteen categories are obtained, including apple core, cylindrical battery, button battery, book, capsule, pencil, toothbrush, trousers, mobile phone, remote control, t-shirt, vegetable leaf, and watermelon peel. The number of samples is counted after the information labeling was completed, and a total of 7671 valid samples are obtained. Some of the garbage image types in the dataset are shown in Fig. 9.

The thirteen subcategories were divided into four major categories according to the existing garbage classification standards in rural areas, including: domestic garbage, recyclable garbage, hazardous garbage and other garbage, to build a garbage dataset containing multiple categories. As seen in Table 4, domestic garbage includes the apple core, toothbrush, vegetable leaf and watermelon peel, with a total of 2204 samples. Recyclable garbage includes the book, trousers, and t-shirt, with a total of 1805 samples. Hazardous garbage includes the battery, button battery, pencil, mobile phone, with a total of 2179 samples. Other garbage includes the capsule and remote control, with a total of 1483 samples. According to the ratio of 7:2:1, the dataset is divided into three parts: training, validation and test sets for comparison experiments and ablation experiments and the training and testing of the model.

**Table 4:** Summary of the dataset.

Garbage category	Target	Number	Total
Domestic garbage	Apple core	658	2204
	Toothbrush	480	
	Vegetable leaf	555	
	Watermelon peel	511	
Recyclable garbage	Book	751	1805
	Trousers	492	
	T-shirt	562	
Hazardous garbage	Battery	558	2179
	Button battery	562	
	Pencil	537	
	Mobile phone	522	
Other garbage	Capsule	684	1483
	Remote control	799	

### Model training and testing

#### Experimental results of introducing the attention combination mechanism

We introduce an attention combination mechanism in the backbone network of the YOLOv5s network model. Then, we iteratively train and test the performance of the YOLOv5s network model with the introduced attention combination mechanism against the original YOLOv5s network model based on the garbage dataset introduced in the “Dataset” section. The classification accuracy of the model is shown in Table 5, and the test results of the model are shown in Table 6.

**Table 5:** Classification accuracy of the proposed YOLOv5s-CS model and YOLOv5s model for training the garbage dataset.

	Target	YOLOv5s			YOLOv5s-CS		
		Precision	Recall	mAP@0.5	Precision	Recall	mAP@0.5
Domestic garbage	Apple core	0.991	0.998	0.997	0.99	0.998	0.997
	Toothbrush	0.891	0.779	0.86	0.985	0.881	0.950
	Vegetable leaf	0.984	0.919	0.974	0.999	0.883	0.987
	Watermelon peel	0.746	0.798	0.809	0.911	0.771	0.826
Hazardous garbage	Battery	0.933	0.854	0.891	0.94	0.894	0.847
	Pencil	0.875	0.872	0.875	0.972	0.92	0.992
	Button battery	0.952	0.941	0.967	0.98	0.961	0.992
	Mobile phone	0.955	0.809	0.944	0.987	0.977	0.976
Recyclable garbage	Book	0.869	0.792	0.852	0.923	0.835	0.886
	Trousers	0.966	0.999	0.995	0.982	0.973	0.935
	T-shirt	0.969	0.942	0.969	0.981	0.982	0.995
Other garbage	Capsule	0.924	0.86	0.922	0.986	0.989	0.975
	Remote control	0.782	0.923	0.887	0.999	0.962	0.928

**Table 6:** Results of network performance testing of the network model with the original network model by introducing the attention combination mechanism.

Method	Precision (%)	Recall (%)	mAP@0.5 (%)	Inference time (ms)	FPS	Model size (MB)	Calculation volume
YOLOv5s	91	88.1	91.8	19.8	32.25	14.4	16.4 GFLOPS
YOLOv5s-CS	94.5	89	95	8.5	62.5	14.1	6.0 GFLOPS

As shown in Table 6, the mAP@0.5 of the YOLOv5s-CS network model reaches 95%, the inference time is only 8.5 ms, the FPS is 62.5 frames/s, and the calculation amount is only 6.0 GFLOPS. Compared to the original YOLOv5s network model, mAP@0.5 is improved by 3.2%, the inference time is reduced by 11.3 ms, and the computation amount is decreased by 10.4 GFLOPS. The introduction of the attention combination mechanism not only improves the detection accuracy but also reduces the complexity of the model and improves the detection speed of the model.

To verify the effectiveness of the proposed attention combination mechanism, we conduct ablation experiments on two attention modules. The experimental results are shown in Table 7. It can be seen from Table 7 that after the introduction of the CBAM attention module, the calculation amount of the model has changed, and it is only 5.9 GFLOPS. At the same time, the model also performs well in terms of inference time, the accuracy rate is improved by 1.1%, and the goal of being lightweight is achieved. After the SE-Net attention module is installed at the end of the backbone network, the accuracy rate of the model reaches 93.6%, which strengthens the feature extraction ability of the model for the target area.

**Table 7:** Attention combination mechanism ablation experiment.

	Precision (%)	Recall (%)	mAP (%)	Inference time (ms)	Calculation volume
YOLOv5s	91	88.1	91.8	19.8	16.4 GFLOPS
YOLOv5s + CBAM	91.6	89	92.9	8.6	5.9 GFLOPS
YOLOv5s + SE-Net	92.1	89.6	93.6	18.9	16.1 GFLOPS
YOLOv5s-CS	94.5	89	95	8.5	6.0 GFLOPS

#### Experimental results of adding a small target detection layer

**Figure 10:**
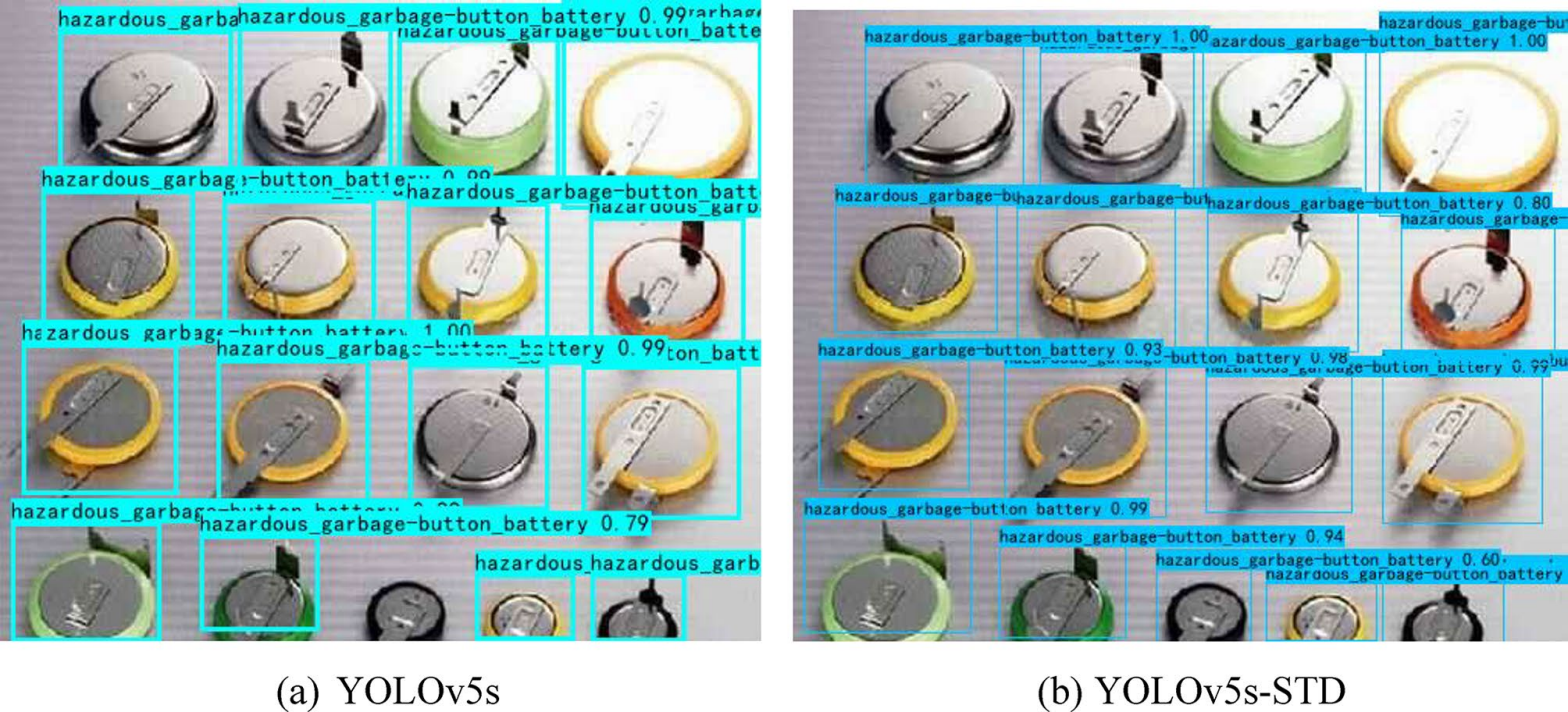
The actual detection effect of the network model.

We add a small target detection layer to the thirty-first layer of the head network to improve the network structure of the model based on the original YOLOv5s network model for detecting objects with small targets. To improve the detection accuracy of the model, a total of four detection layers for the output feature maps are used, which are able to detect smaller target objects. The classification accuracy of the model is shown in Table 8, and the test result of the model is shown in Table 9. Figure 10 shows the actual test results of the model.

**Table 8:** Classification accuracy of the proposed YOLOv5s-STD model and YOLOv5s model training garbage dataset.

	Target	YOLOv5s			YOLOv5s-STD		
		Precision	Recall	mAP@0.5	Precision	Recall	mAP@0.5
Domestic garbage	Apple core	0.991	0.998	0.997	0.99	0.998	0.997
	Toothbrush	0.891	0.779	0.86	0.985	0.881	0.950
	Vegetable leaf	0.984	0.919	0.974	0.999	0.883	0.987
	Watermelon peel	0.746	0.798	0.809	0.911	0.771	0.826
Hazardous garbage	Battery	0.933	0.854	0.891	0.94	0.894	0.847
	Pencil	0.875	0.872	0.875	0.972	0.92	0.992
	Button battery	0.952	0.941	0.967	0.98	0.961	0.992
	Mobile phone	0.955	0.809	0.944	0.987	0.977	0.976
Recyclable garbage	Book	0.869	0.792	0.852	0.923	0.835	0.886
	Trousers	0.966	0.999	0.995	0.982	0.973	0.935
	T-shirt	0.969	0.942	0.969	0.981	0.982	0.995
Other garbage	Capsule	0.924	0.86	0.922	0.986	0.989	0.975
	Remote control	0.782	0.923	0.887	0.999	0.962	0.928

**Table 9:** Addition of the network model of a small target detection layer and the network performance test results of the original network model.

Method	Precision (%)	Recall (%)	mAP@0.5 (%)	Inference time (ms)	FPS	Model size (MB)	Calculation volume
YOLOv5s	91	88.1	91.8	19.8	32.25	14.4	16.4 GFLOPS
YOLOv5s-STD	93.6	90.2	93.6	32.2	18.36	15.2	27.7 GFLOPS

As shown in Table 9, the mAP@0.5 of the YOLOv5s-STD network model reaches 93.6%, a 1.8% improvement of mAP@0.5 compared to the original YOLOv5s network model. From Fig. 10, there are no missed detection behaviors when using the YOLOv5s-STD network model, and all the target objects are detected. However, the original YOLOv5s network model has missed target object behavior when detecting multiple and small targets. Increasing the small target detection layer not only improves the detection accuracy of the model but also solves the problem of the original model in detecting small and multiple targets.

#### Experimental results using the CIoU loss function

**Figure 11:**
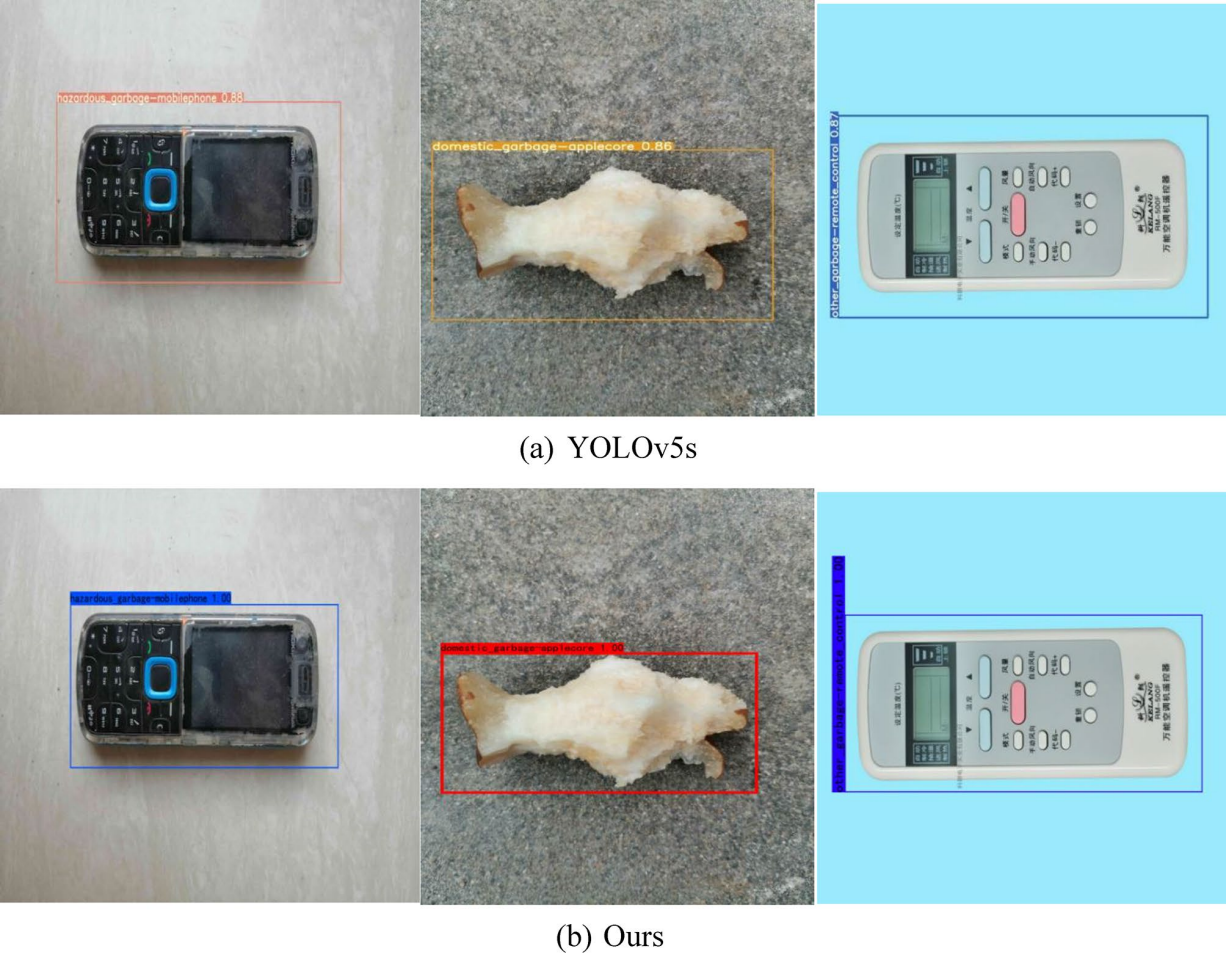
Prediction frame detection effect comparison.

The YOLOv5s network model uses the GIoU loss function by default. There are two problems in the GIoU loss function. When the GIoU degenerates into IoU in the prediction bounding box and the ground truth bounding box and when the prediction results are output, there is still a low similarity between the prediction bounding box and the ground truth bounding box. Therefore, we replaced the GIoU loss function with the CIoU loss function based on the YOLOv5s network model. When outputting the prediction result, the prediction bounding box is made more consistent with the ground truth bounding box of the target object. The experimental test result is shown in Fig. 11.

The actual detection effect in Fig. 11 shows that the network model with the CIoU loss function is more consistent with the ground truth bounding box of the target object in the output prediction result compared with the YOLOv5s network model. The problem that the GIoU loss function does not have a high similarity between the predicted bounding box and the ground truth bounding box when outputting the prediction results is solved, and the predicted bounding box is optimized.

#### Adam optimizer training results

**Figure 12:**
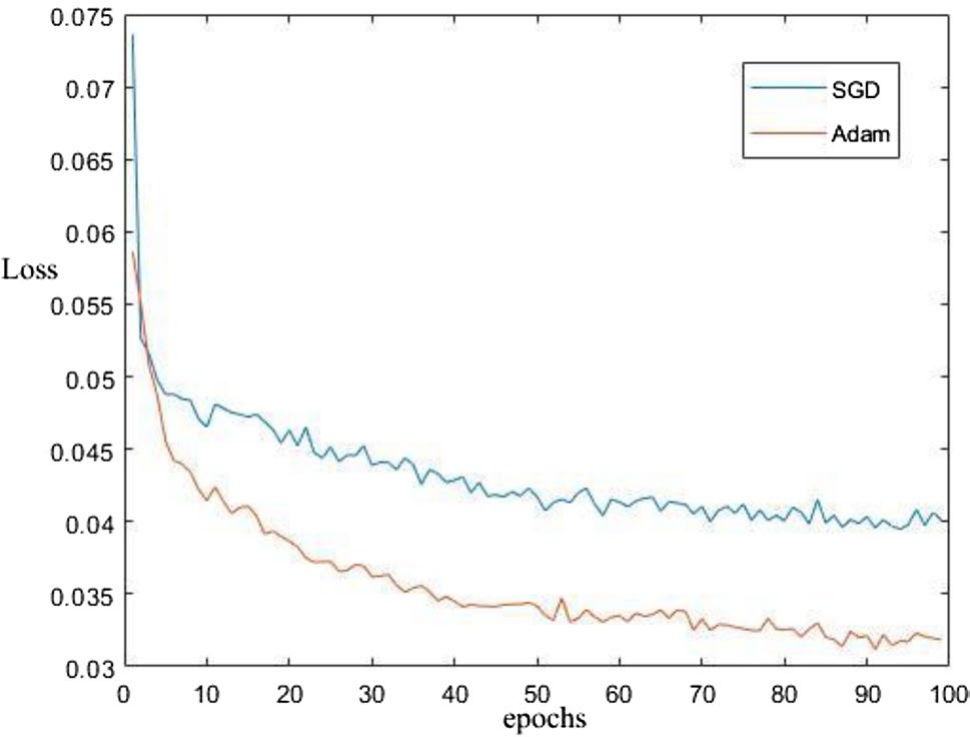
The training results of the SGD optimization algorithm and Adam optimization algorithm for the loss value in the YOLOv5 network model.

The YOLOv5s network model uses the SGD optimization algorithm by default. The SGD optimization algorithm has two problems when training the network model: (1) the slow convergence of the loss function and (2) the large fluctuation of loss values. Therefore, we use the Adam optimization algorithm to train the YOLOv5s network model with the characteristics of fast convergence and adaptive updating of neural network weights. The model training result is shown in Fig. 12.

Where the horizontal coordinate represents the number of iterations of the training model, and the vertical coordinate represents the value of the loss function of the model. The red curve represents the model trained with the Adam optimization algorithm, and the blue curve represents the model trained with the SGD optimization algorithm. As seen from Fig. 12, the red curve is somewhat better than the blue curve in terms of declining speed and smoothness. This indicates that the model trained based on the Adam optimization algorithm outperforms the original network model in terms of the convergence speed and the degree of loss value fluctuation.

#### Experimental results of related improvements

**Figure 13:**
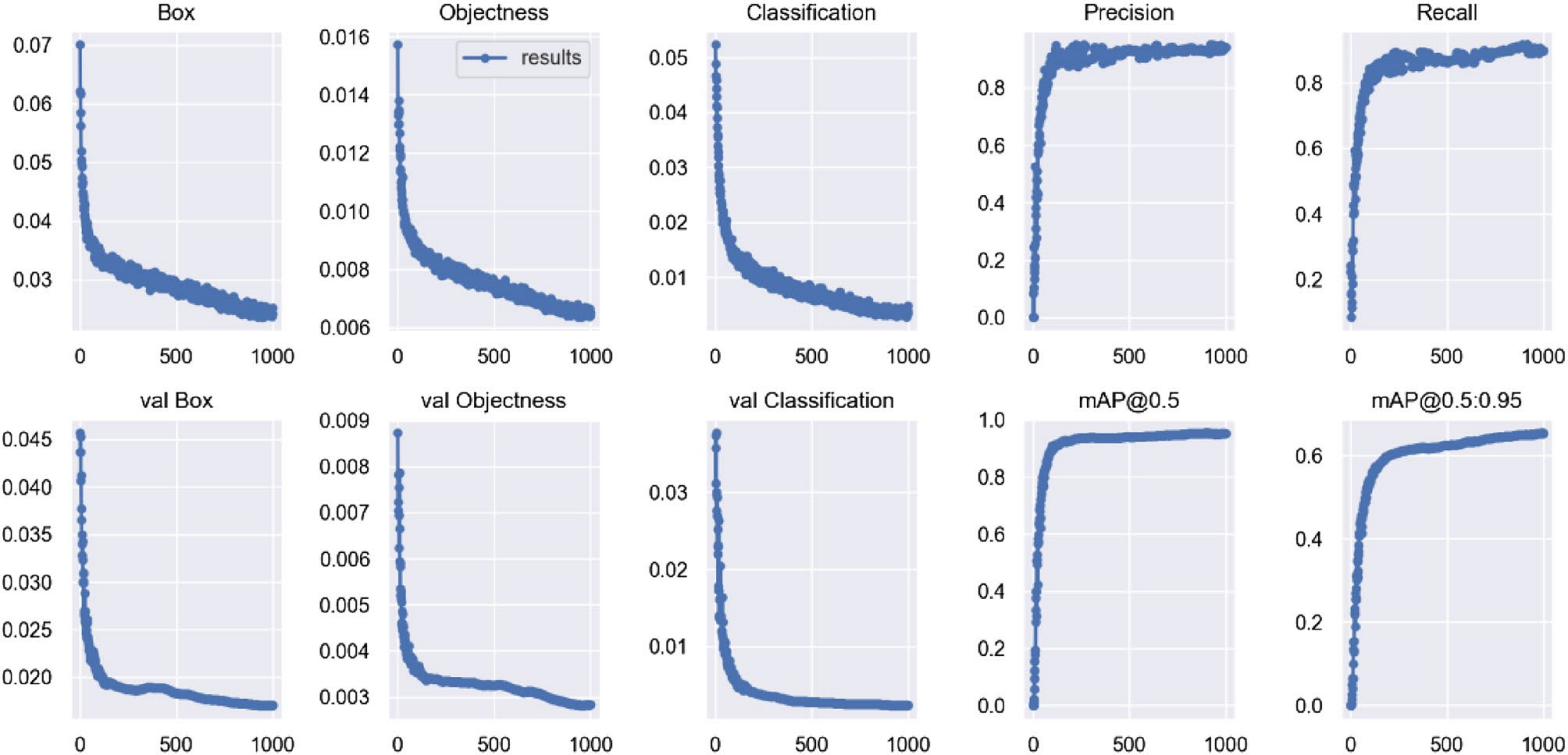
Record of the YOLOv5s-CSS network model training process.

**Figure 14:**
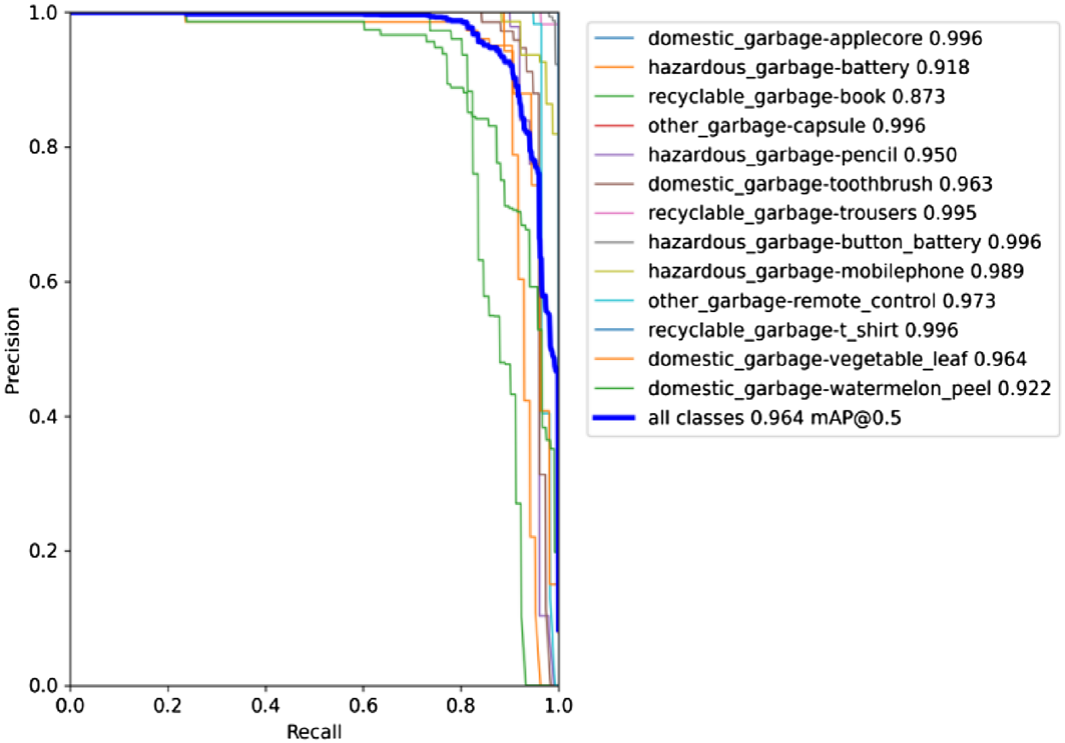
Accuracy rate curve of the YOLOv5s-CSS network model.

**Figure 15:**
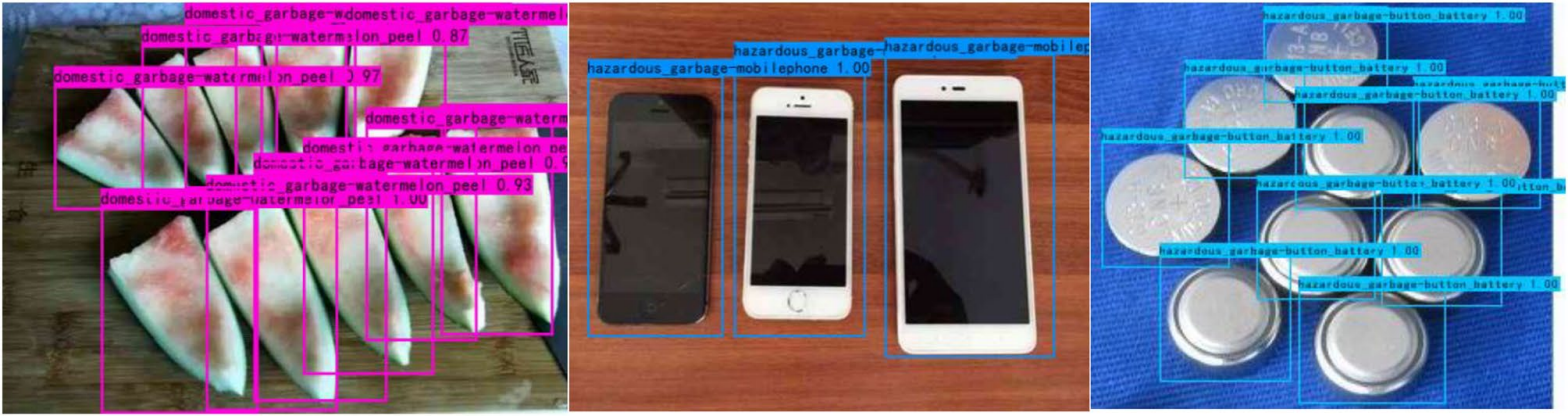
Images from the test dataset show the results of garbage detection.

We introduce an attention combination mechanism at the backbone network side based on the YOLOv5s network model, add a small target detection layer to the head network, use the CIoU loss function to replace the GIoU loss function, and selecte the Adam optimization algorithm to train the improved network model and name it YOLOv5s-CSS. The classification accuracy of the model is shown in Table 10, and the test results of the model are shown in Table 11. Figure 13 shows the training process of the model. Figure 14 shows the accuracy rate curve of the model, and Fig. 15 shows the detection effect of the model.

**Table 10:** Classification accuracy of the proposed YOLOv5s-CSS model and YOLOv5s model training garbage dataset.

	Target	YOLOv5s			YOLOv5s-CSS		
		Precision	Recall	mAP@0.5	Precision	Recall	mAP@0.5
Domestic garbage	Apple core	0.991	0.998	0.997	0.99	0.998	0.997
	Toothbrush	0.891	0.779	0.86	0.985	0.882	0.964
	Vegetable leaf	0.984	0.919	0.974	0.999	0.884	0.97
	Watermelon peel	0.746	0.798	0.809	0.91	0.771	0.899
Hazardous garbage	Battery	0.933	0.854	0.891	0.939	0.894	0.942
	Pencil	0.875	0.872	0.875	0.971	0.92	0.963
	Button battery	0.952	0.941	0.967	0.978	0.961	0.994
	Mobile phone	0.955	0.809	0.944	0.987	0.977	0.995
Recyclable garbage	Book	0.869	0.792	0.852	0.921	0.835	0.866
	Trousers	0.966	0.999	0.995	0.982	0.979	0.995
	T-shirt	0.969	0.942	0.969	0.981	0.982	0.996
Other garbage	Capsule	0.924	0.86	0.922	0.986	0.989	0.995
	Remote control	0.782	0.923	0.887	0.999	0.963	0.971

**Table 11:** Network performance test results of the proposed YOLOv5s-CSS network model and the original network model.

Method	Precision (%)	Recall (%)	mAP@0.5 (%)	Inference time (ms)	FPS	Model size (MB)	Calculation volume
YOLOv5s	91	88.1	91.8	19.8	32.25	14.4	16.4 GFLOPS
YOLOv5s-CSS	93.5	91.1	96.4	13.1	47.6	15.8	8.9 GFLOPS

It can be seen from Table 11 that the performance of the YOLOv5s-CSS network model mAP@0.5 is 96.4%; compared with the original YOLOv5s network model, the mAP@0.5 is increased by 4.6%, FPS is increased by 47.6%, inference time is reduced by 6.7 ms, and the amount of calculation is reduced by 84.2%.

The YOLOv5s-CSS network model can detect and classify the garbage in the image and achieve the real-time detection effect and still has excellent performance in the face of multiple and small targets.

#### Experimental results of fuzzy scenes

**Figure 16:**
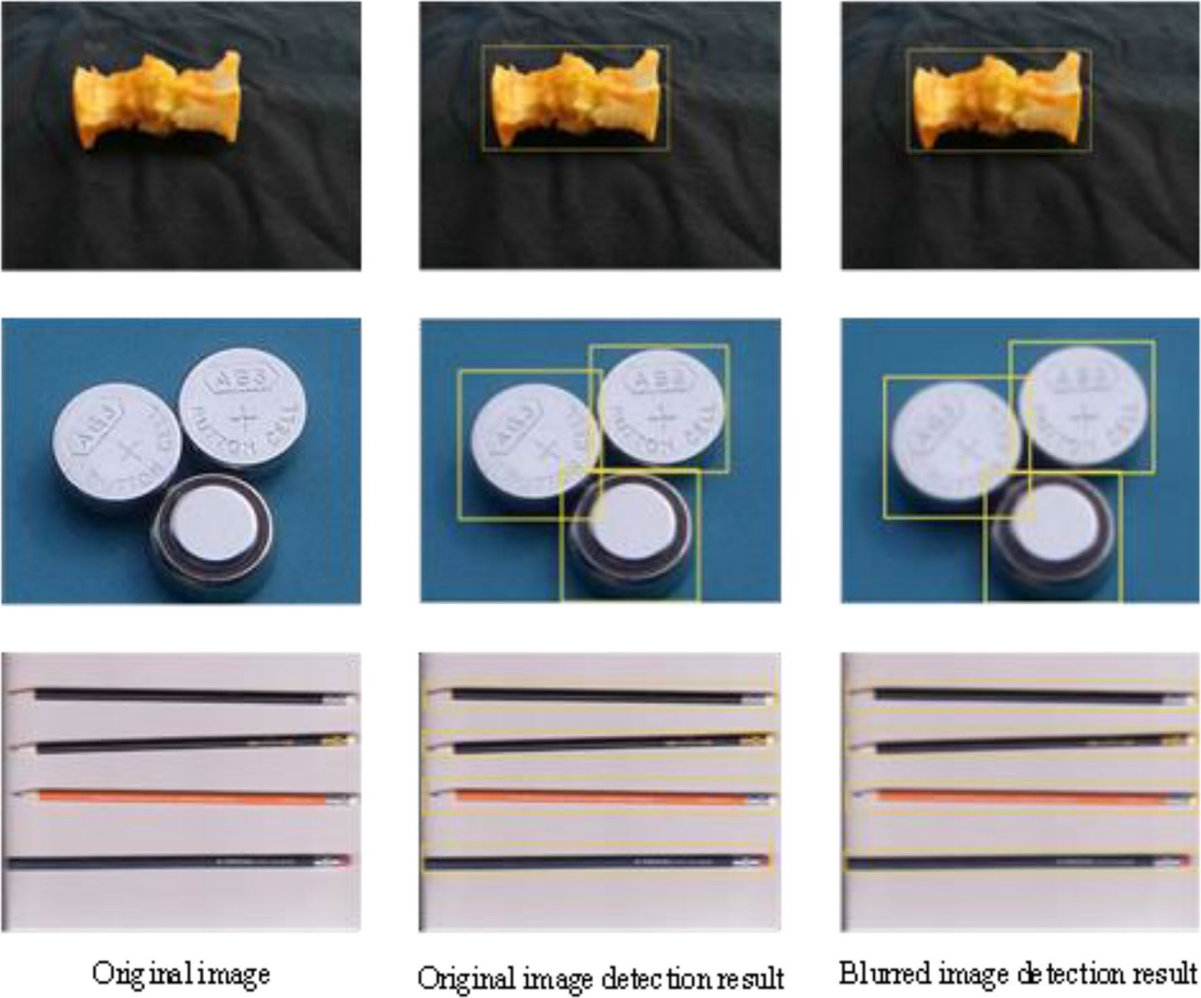
The first column represents the original image, the second column represents the detection effect of the original image, and the third column represents the detection effect of the blurred image.

When the detected object is in a certain blurred state, it tends to reduce the detection effect of the model. However, The YOLOv5 algorithm itself also adopts the Mosaic data enhancement method on the input side. The Mosaic data enhancement is a data enhancement method proposed by the YOLOv5 algorithm for blurred images at the input end. The method stitches images by random scaling, random cropping, and random arrangement, and has a certain enhancement effect on the detection effect of blurred images. The detection effect in the fuzzy state is shown in Fig. 16.

As can be seen in Fig. 16, the method proposed in this study can still accurately detect all target objects even though the images are in a blurred state. This is because although the image will exist in a blurred and unclear state, the deep feature information about the target object image itself still exists.

#### Experimental results of unbalanced classes in dataset

We targeted thirteen sample categories in the dataset as the subjects of our research, and the experimental procedure took a control variable approach. The sample sizes of ‘vegetable leaf’ and ‘mobile phone’ were reduced by 63.9 and 80.8%, respectively, to bring them to a state of sample category imbalance. We iteratively train and record the detection accuracy of the proposed model in this study when the sample categories are in an unbalanced state. The experimental results are shown in Table 12.

**Table 12:** Sample distribution imbalance model test results.

	Number	AP (%)	mAP (%)	Number	AP (%)	mAP (%)
Applecore	658	99.7	90.3	658	99.7	96.4
Vegetable leaf	200	89.1		555	98.7	
Mobile phone	100	86.7		522	97.6	
Toothbrush	480	96.4		480	96.4	
Watermelon peel	511	89.9		511	89.9	
Book	751	86.6		751	86.6	
Trousers	492	99.5		492	99.5	
T-shirt	562	99.6		562	99.6	
Battery	558	94.2		558	94.2	
Button battery	562	99.4		562	99.4	
Pencil	537	96.3		537	96.3	
Capsule	684	99.5		684	99.5	
Remote control	799	97.1		799	97.1	

As can be seen from Table 12, if the number of samples and classes are unbalanced during the training process, this will lead to a reduction in the performance metrics of the training model. The best performance tends to be obtained when the classes are balanced, while class imbalance tends to reduce the effectiveness of the model. If the training samples are unbalanced, the balanced samples can usually be oversampled before training. This conclusion also applies to other classical target detection algorithms.

### Comparison with mainstream detection algorithms

**Figure 17:**
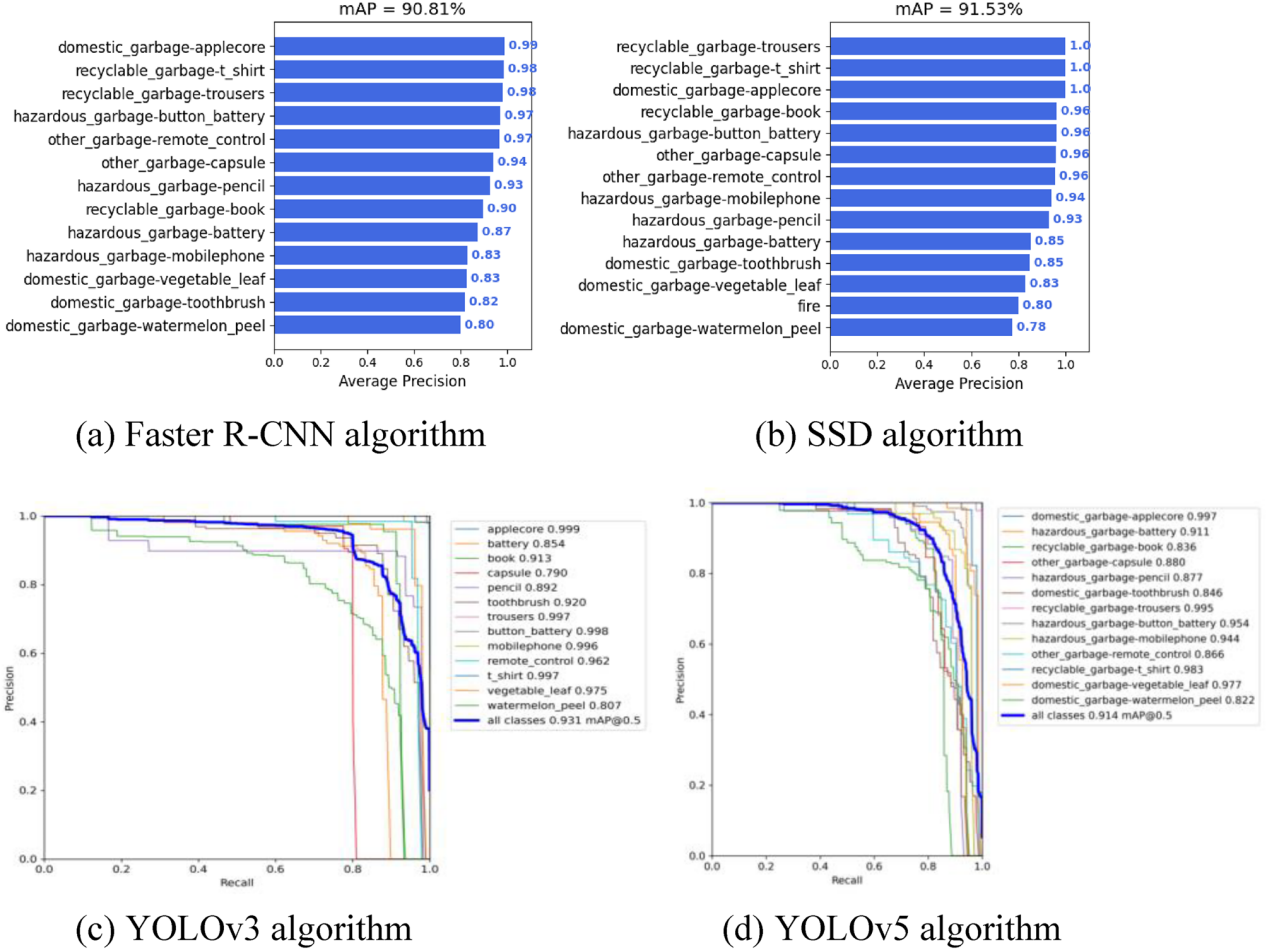
The mAP training results of the four detection algorithms.

In the field of deep learning, mainstream target detection algorithms include the Faster R-CNN, SSD and YOLO series algorithms. We perform comparison experiments on the Faster R-CNN, SSD, YOLOv3^35^, YOLOv5 and YOLOv5s-CSS algorithms based on the garbage dataset in Section “Dataset”. The experimental procedure is shown in Fig. 17, and the experimental results are shown in Table 13.

**Table 13:** Model test performance between different detection algorithms.

Method	Faster RCNN	SSD	YOLOv3	YOLOv5s	YOLOv5s-CSS
mAP@0.5	90.81%	91.53%	93.1%	91.8%	96.4%
FPS	10.9	10.2	8.85	32.25	47.6

It can be inferred from Table 13 that the YOLOv5s-CSS network model has a higher mAP value and detection speed compared to different detection algorithms. While improving the detection accuracy, it is also able to meet the requirements of real-time detection.

In Table 14 we can see the comparison of the detection results of the proposed models from the literature. We can see that most of the models are based on transfer learning and complex two-stage detection algorithms. This leads to a reliance on the validity of the transferred model while increasing the computational cost. The idea is to train a lightweight garbage model that does not depend on other models that perform well. Therefore, we can implement it on most devices and run it without problems. If we compare metrics, our proposed architecture achieves the best results in the presented categories. This provides yet another proof of the efficiency of our solution.

**Table 14:** Comparison of our developed model with other garbage detection methods in the literature.

	Year	Method	Accuracy (%)
Our model	2022	YOLOv5s + CBAM + SE-Net + CIoU + Adam	96.4
He et al.^36^	2021	ResNet50 Network and Migration Learning	91.42
Aleen et al.^37^	2021	Faster RCNN and Migration Learning	96
Middya et al.^38^	2021	Faster RCNN and InceptionV2	92
Verma et al.^39^	2022	CNN model	94
Assis et al.^40^	2021	YOLOv3 model	93.41
Sunny et al.^41^	2019	ALexNet model	96
Zhi et al.^42^	2020	Training SqueezeNet using transfer learning techniques	87.7

## Discussion

In this paper, an intelligent detection method for rural garbage is proposed. To meet the needs of rural garbage detection, we conduct in-depth research in the field of garbage classification based on the YOLOv5 algorithm. The proposed YOLOv5s-CSS model is constructed based on the YOLOv5s model with an attention composition mechanism, a small object detection layer and a CIoU loss function. When training the model, we solve the problem of low model convergence speed and large fluctuation of loss value by adopting the Adam optimization algorithm.

To validate the proposed method to build a fast and accurate multilevel deep learning model, we compare the classical object detection algorithms with the state-of-the-art garbage detection methods. The final experimental results show that the proposed YOLOv5s-CSS model achieves 93.5%, 91.1%, 96.4% and 47.6 frames/s in terms of precision, recall, mAP value and FPS, respectively.

From Table 11, it can be concluded that compared with the YOLOv5s model, the proposed YOLOv5s-CSS model improves the inference speed by 6.7 ms, the detection accuracy by 4.6%, and reduces the computational load of the model. It can be seen from Table 13 that compared with the classic target detection algorithms Fast R-CNN, SSD and YOLOv3, the improved algorithm has a higher FPS value and mAP@0.5 value, which are 47.6 frames/sec and 96.4%, respectively. The accuracy and real-time performance are excellent. The detection results comparison of the proposed models can be seen from the literature^36,42^. Our proposed method has higher accuracy and outperforms the current state-of-the-art garbage detection algorithms. In Table 14, most of the models are based on transfer learning and a complex two-stage detection algorithm. This leads to relying on the effectiveness of the transport model while increasing the computational cost. The idea is to train a lightweight garbage model that does not depend on other well-behaved models. The detection speed of the proposed YOLOv5s-CSS model is 0.021 s, and the calculation amount of the model is only 8.9 GFLOPS, which meets the needs of most devices.

## Conclusion and future work

Deep learning techniques have achieved significant performance in the field of garbage classification. Compared with traditional garbage classification technology, deep learning methods can improve the performance and detection accuracy of garbage classification, but they still have challenges. We propose the following four guiding opinions based on our research: (1) conduct experimental comparisons with mainstream detection algorithms on garbage dataset to select the detection algorithm and network model suitable for the study; (2) improve the detection capability of the network model for small and multiple targets; (3) reduce the complexity of the network model and focus on improving the real-time performance of the network model detection; and (4) optimize the detection effect of the output prediction bounding box. To test these ideas, four improved methods have been proposed to build fast and accurate multilevel deep learning models for identifying and classifying common garbage in our lives. The final experimental results verify the effectiveness of our model designed based on these four guiding ideas. Compared with classical target detection algorithms and mainstream detection algorithms, the proposed YOLOv5s-CSS model not only satisfies the faster detection speed requirement but also greatly improves the detection accuracy, which can meet the requirements of real-time detection of domestic garbage. It reduces the complexity of the network model to a certain extent, and has excellent performance for small target and multitarget detection.

Although this study has proven the effectiveness of the proposed method through analysis and experiments, there are still some limitations, mainly in two aspects: (1) When the sample data are small or unbalanced, the model's performance will be affected, reducing the effectiveness of the model. (2) In practical applications, with the development of the times, an increasing number of garbage types will be generated, and the proposed model needs to be updated in real time so that more types of garbage can be detected.

In future research, we will attempt to embed this system into mobile devices. Therefore, we can build a smart garbage sorting infrastructure in rural areas, so that the detection results can be shared in real time through device records, allowing developers to update methods in a timely manner and classify and detect garbage more efficiently. This reduces the consumption of labor costs and accelerates the development of intelligent garbage detection.

## Data Availability

The data provided in this study can be obtained from the corresponding author J.X.
